# Quantification of left ventricular trabeculae using cardiac magnetic resonance imaging for the diagnosis of left ventricular non-compaction

**DOI:** 10.1186/1532-429X-17-S1-P301

**Published:** 2015-02-03

**Authors:** Yeonu Choi, Yeon Hyeon Choe, Sung Mok Kim, Sang-Chol Lee, Sung-A Chang

**Affiliations:** 1 Sungkyunkwan Univerity School of Medicine, Seoul, Korea (the Republic of; 2 Cardiovascular Stroke Imaging Center, Samsung Medical Center, Sungkyunkwan University School of Medicine, Seoul, Korea (the Republic of

## Background

Left ventricular non-compaction (LVNC) is an unclassified cardiomyopathy and there is no consensus in the diagnosis of LVNC. The aims of this study were to establish quantitative method to diagnose isolated LVNC (INC) using cardiac magnetic resonance (CMR) imaging and to suggest novel qualitative method to diagnose isolated LVNC.

## Methods

This retrospective study included 145 subjects with moderate to severe trabeculation of LV myocardium [24 patients with isolated LVNC, 33 patients with non-isolated LVNC, 30 patients with dilated cardiomyopathy (DCM) with noncompaction, 27 patients with DCM and 31 healthy control subjects]. LVNC patients had to fulfill Petersen's CMR criteria. LV ejection fraction, global LV volume, trabeculated LV volume, and number of segments with late gadolinium enhancement were measured. And most prominent noncompacted (NC), compacted (C), normal mid-septum, normal mid-lateral wall and apical trabeculation thickness on the end-diastolic frames of each long-axis slice was also measured.

## Results

*Results* In the patients with isolated LVNC, the percentage of trabeculated LV volume was 1.4 times higher (42.6 ± 13.8) than in DCM (30.3 ± 14.3, p<0.0001)[YHC1], and 1.7 times higher than in controls (24.8 ± 7.1, p<0.0001). However, there were no significant differences between INC and DCMNC (47.1 ± 17.3, p = 0.210). And a value of percentage of trabeculated LV volume above 32% was predictive of isolated LVNC with a specificity of 90.3% (CI, 74.2-98.0%) and sensitivity of 79.2% (CI, 57.8-92.9%). A value of NC/septum over 1.1 was considered predictive for isolated LVNC with a specificity of 80.6% (CI, 62.5-92.5%) and sensitivity of 95.8% (CI, 78.9-99.9%). And a value of apex/C above 3.1 was considered predictive of isolated LVNC with a specificity of 93.5% (CI, 78.6-99.2%) and sensitivity of 87.5% (CI, 67.6-97.3%).

## Conclusions

As a quantitative approach, a trabeculated LV volume above 32% of the total LV volume is diagnostic for isolated LVNC with high sensitivity and specificity. And as a qualitative approach, apex/C and NC/septum ratio could be useful for supplemental diagnostic criteria.

## Funding

None.

